# Neurological Features and Their Association With Gender in Diabetes Mellitus Patients

**DOI:** 10.7759/cureus.39687

**Published:** 2023-05-30

**Authors:** Muhammad Mubeen, Ahsan Masood, Muhammad Ulusyar Khan, Hira Khalid Chohan, Aisha Jamal, Musarat Khalid Chohan, Sadaf Sattar Abbassey, Adnan Anwar, Atif A Hashmi

**Affiliations:** 1 Internal Medicine, Dow University of Health Sciences, Karachi, PAK; 2 Internal Medicine, Gomel State Medical University, Gomel, BLR; 3 Internal Medicine, Bolan University of Medical and Health Sciences, Quetta, PAK; 4 General Surgery, Dow University of Health Sciences, Karachi, PAK; 5 Internal Medicine, Army Medical College, Rawalpindi, PAK; 6 Anaesthesiology, Jinnah Sindh Medical University, Karachi, PAK; 7 Family Medicine, Sindh Services Hospital, Karachi, PAK; 8 Physiology, Hamdard College of Medicine and Dentistry, Karachi, PAK; 9 Internal Medicine, Essa General Hospital, Karachi, PAK; 10 Pathology, Liaquat National Hospital and Medical College, Karachi, PAK

**Keywords:** type 1 diabetes, diabetes type 2, gender, swelling and burning, tingling or numbness

## Abstract

Introduction

Understanding the wide range of clinical signs and symptoms associated with diabetes mellitus (DM) is crucial because people with DM are frequently misdiagnosed, given incorrect care, or poorly controlled. Therefore, the purpose of this study was to evaluate the neurological symptoms associated with type 1 and type 2 DM patients with respect to patient gender.

Methods

This was a cross-sectional multicenter study that was conducted at different hospitals using a non-probability sampling method. The duration of the study was eight months, from January 2022 to August 2022. The study involved 525 type 1 and type 2 DM patients with an age range from 35 to 70 years. Demographic details such as age, gender, socioeconomic status, past medical history, presence of comorbidities, type, and duration of DM, and neurological features were recorded as frequencies and percentages. A Chi-square test was used to determine the association between neurological symptoms associated with type 1 and type 2 DM and gender.

Results

The study findings showed that of 525 diabetic patients, 210 (40.0%) were females and 315 (60.0%) were males. The mean male and female mean ages were 57.36±14.99 and 50.52±14.8 years, respectively, with a significant difference with respect to gender (p<0.001). The prevalence of neurological manifestations showed that irritability or mood swings were reported by most of the male 216 (68.6%) and 163 (77.6%) female diabetic patients, with a significant association noticed (p=0.022). Moreover, a significant association was observed between both genders in terms of swelling of feet, ankles, hands, and eyes (p=0.042), confusion or difficulty in concentration (p=0.040), burning pain in feet or legs (p=0.012), and muscular pain or cramps in legs or feet (p=0.016).

Conclusion

This study concluded that the prevalence of neurological manifestations was high among diabetic patients. Most of the neurological symptoms were significantly more pronounced in female diabetic patients. Moreover, most of the neurological symptoms were associated with the type (type 2 DM) and duration of DM. The presence of hypertension, dyslipidemia, and smoking also influenced some neurological manifestations.

## Introduction

Diabetes mellitus (DM) is the leading global disease in both advanced and developing nations and creates a dilemma for health [1]. In 2019, the incidence of DM is expected to be 9.3% globally [2]. In 2030 and 2045, respectively, there will be a rise in the prevalence of DM, with estimates of 578 million cases (10.2%) and 700 million cases (10.9%) [2]. DM is an endocrine disease that is defined by poor blood glucose control, primarily by a persistent state of hyperglycemia along with periodic episodes of hypoglycemia. The most prevalent types of DM are type 1 and type 2; however, there are also other subtypes that can be caused by endocrine disorders, medications, infections, and immunologic, inherited, and pancreatic factors. The quality of life can be negatively impacted by these metabolic imbalances, which are the primary cause of death and can result in a number of issues affecting the kidneys, heart, blood vessels, eyes, and neurological system [3,4]. DM complications can be divided into two groups: macrovascular and microvascular complications. The most frequent neuropathy and one of the microvascular consequences of diabetes that result from persistently inadequate glucose control is diabetic neuropathy [5].

The brain accounts for 2% of our total body weight and uses 25% of body oxygen and 20% of blood glucose. The hypothalamus works in conjunction with several hormones to regulate dietary intake, energy consumption, the secretion of insulin, the production of glucose by the liver, and glucose/fatty acid metabolism in fat tissue and skeletal muscle [6,7]. The energy essential for the brain to function ideally, from cellular upkeep to the production of neurotransmitters, is provided by glucose. In addition to contributing to the pathogenesis of neurological illnesses, glucose plays a critical role in the control of oxidative stress, cellular death, and pathways whose processes are involved in disturbed hypothalamic pathways and monitoring of glucose and insulin [8,9].

Complications of DM, and the morbidity and mortality associated with DM are a major cause of healthcare burden [1]. Higher diabetes complications, which have a major impact on patients' living standards, are linked to higher DM prevalence [10]. Typically, diabetic peripheral neuropathy (DPN) is described as a symmetrical, persistent sensory polyneuropathy that is length dependent. There are various paths for DPN; some have painful manifestations, while others do not [11].

Despite being the most frequent diabetes complication, DPN is usually underdiagnosed [12]. Additionally, there is a gap in accurately assessing the prevalence of DPN, which leads to a significant variation in prevalence reports [13]. In Saudi Arabia, the findings of Wang et al. reported that the occurrence of DPN was determined to be 19.9% [14]. Similarly, Algeffari observed that the incidence of painful DPN in type 2 diabetes was predicted to be 35% and had poor treatment adherence. Furthermore, it was discovered that elevated hemoglobin levels were connected with painful DPN [15].

Generally, DPN is one of the common and severe microvascular consequences of diabetes [16]. As it is a significant cause of peripheral nerve injury, DPN lowers life expectancy and increases the likelihood of impairment in people with diabetes [17]. Controversy exists in the frequency and risk factors of DPN globally, particularly in low-and middle-income nations.

Uncomfortable dysaesthesia or discomfort in the feet and lower legs are common in patients with newly identified or poorly managed diabetes, but these symptoms usually subside quickly after euglycemia is established. Diabetic sensory polyneuropathy poses a significant risk for the occurrence of plantar ulcers owing to the loss of defensive perception [18]. Literature on the different relationships between hyperglycemia and its consequences on the neurological system is scarce in Pakistan. Therefore, this study assessed the frequency and association of neurological symptoms among patients with type 1 and type 2 diabetes.

## Materials and methods

This was a cross-sectional multicenter study that was performed at different hospitals using a non-probability sampling method. Ethical approval was obtained from Essa General Hospital (Essa/32/2023). The data were collected prospectively using a pre-designed questionnaire at outpatient clinics. Written informed consent was obtained from all participants. The study duration comprised eight months from January 2022 to August 2022. Only diagnosed cases of DM were included in the study. The study involved 525 type 1 and type 2 diabetes patients ranging from 35 to 70 years, whereas patients with intense weight loss, low glucose tolerance, low fasting glucose, any surgical treatment, and patients who underwent chemotherapy were excluded from the study. In addition, cases with inadequate past medical information or who refused to participate were excluded from the study.

Demographic details of type 1 and type 2 diabetes patients, such as gender, age, socioeconomic status, past medical history, presence of comorbidities, type and duration of diabetes, and neurological features, were recorded. The latest glycated hemoglobin (HbA1c) level was used to identify patients with both types of DM. Additionally, body mass index (BMI) was calculated by measurement of height and weight. The presence of stress, anxiety, and depressive symptoms was also assessed. Researchers took measurements of heart rate, breathing rate, and blood pressure. The average of the three readings' pulse rates was determined by the maximum blood pressure after three measures. A questionnaire was used to collect data on current medical history, neurological signs, and symptoms. Additionally, measures of relevant biochemical markers like triglycerides, total cholesterol, high-density lipoprotein cholesterol, and low-density lipoprotein cholesterol were made as well as random blood sugar readings.

The data were analyzed using IBM SPSS Statistics for Windows, Version 26.0 (Released 2019; IBM Corp., Armonk, New York, United States). Means and standard deviations were documented for continuous variables. Demographic factors such as gender and neurological features associated with type 1 and type 2 diabetes were documented as frequencies and percentages. The relationship between neurological manifestations associated with both types of diabetes and gender was determined by the Chi-square test. Likewise, an independent t-test was used to observe the association between means of demographic parameters. A p-value of <0.05 was statistically significant.

## Results

The study included 525 participants with type 1 and type 2 DM. There were 210 (40.0%) females and 315 (60.0%) males among them. The mean ages of the males and females were 57.36±14.99 and 50.52±14.8 years, respectively, with a significant difference between both genders (p<0.001). The mean weights were 71.31±13.72 Kg for males and 63.56±14.38 Kg for females, with a statistically significant difference between the genders (p<0.001). Moreover, the difference in mean height between genders was substantial (p=0.010), measuring 68.75±10.72 inches for males and 66.3±10.56 inches for females. The mean BMIs for males and females were 25.5±11.86 and 23.85±8.49 Kg/m2, respectively, with no gender-related differences (p=0.068). The respiratory rates for men were 19.87±6.03 and for women were 17.52±5.22 breaths/min, with a significant correlation between both genders (p<0.001). The mean temperatures of males and females were 74.51±24.96 and 62.74±25.26oF, respectively, with a significant association between genders (p<0.001). The blood pressure of males and females was 177.01±47.77 and 163.62±49.7 mmHg, respectively, with a significant association between genders (p=0.002), and it was observed that the mean duration of hypertension of males and females was 5.33±4.81 and 4.32±4.09 years, respectively, with a statistically substantial relationship between genders (p=0.010). Additionally, a significant difference in mean heart rates was seen between males and females (87.6±10.84 and 81.3±11.39, respectively, p<0.001). The average daily cigarette consumption for men and women was 3.69±5.44 and 0.33 ±1.69, respectively, with statistically significant gender differences (p<0.001). Males and females had diabetes for 2.07±0.72 and 1.90±0.69 years, respectively, with a statistically significant relationship between both genders (p=0.010). Additionally, there was a statistically significant association between genders (p=0.025) with a male random blood sugar (RBS) of 285.42±106.40 and a female RBS of 265.1±98.01, as shown in Table 1.

**Table 1:** Demographic characteristics of diabetes patients with respect to gender SD: standard deviation *p-value significant as <0.05

Variables	Male	Female	p-value
	Mean±SD	Mean±SD	
Age (years)	57.36±14.99	50.52±14.8	<0.001*
Weight (Kg)	71.31±13.72	63.56±14.38	<0.001*
Height (Inch)	68.75±10.72	66.3±10.56	0.010*
BMI (kg/m^2^)	25.5±11.86	23.85±8.49	0.068
Respiratory rate (breath/min)	19.87±6.03	17.52±5.22	<0.001*
Temperature (^o^F)	74.51±24.96	62.74±25.26	<0.001*
Blood pressure (mm of Hg)	177.01±47.77	163.62±49.7	0.002*
Duration of hypertension (if present)	5.33±4.81	4.32±4.09	0.010*
Heart rate (beats/min)	87.6±10.84	81.3±11.39	<0.001*
Daily cigarette consumption (if smoking history present)	3.69±5.44	0.33±1.69	<0.001*
Duration of diabetes (years)	2.06±0.72	1.90±0.69	0.010*
Random blood sugar	285.42±106.40	265.1±98.01	0.025*

The majority of male diabetic patients 172 (54.6%) and female diabetic patients 140 (66.7%) belonged to the middle class, while 100 (31.7%) male patients and 27 (12.9%) female patients belonged to the high class, with a statistically significant relationship among them (p<0.001). The majority of the male diabetes patients 148 (47.0%) and female diabetes patients 108 (51.4%) had a duration of one to five years, with a significant association between them (p=0.023). As far as the type of diabetes is concerned, mostly male 273 (86.7%) and female patients 150 (71.4%) had type 2 diabetes, with a substantial difference between them (p<0.001). More than half of the diabetic male 216 (68.6%) and female 126 (60.0%) patients had hypertension, with a significant association between both genders (p=0.044). The majority of the male 239 (75.9%) and female diabetic patients 135 (64.3%) showed a history of dyslipidemia, and a significant association was found among them (p=0.004). Additionally, a history of depression was reported by 123 (39.0%) and 57 (27.1%) male and female diabetic patients, respectively, with a substantial relationship between them (p=0.005). Furthermore, there was a significant difference observed between genders in terms of smoking (p<0.001), and physical activity (p<0.001), as shown in Table 2.

**Table 2:** Prevalence of comorbidities, and socioeconomic status with respect to gender DM: diabetes mellitus

Variables		Male	Female	p-value
		n (%)	n (%)	
Socioeconomic status	Low	43 (13.7%)	43 (20.5%)	<0.001*
	Middle	172 (54.6%)	140 (66.7%)	
	High	100 (31.7%)	27 (12.9%)	
Duration of DM	<1 year	73 (23.2%)	61 (29.0%)	0.023*
	1-5 years	148 (47.0%)	108 (51.4%)	
	>5 years	94 (29.8%)	41 (19.5%)	
Type of DM	Type 1	42 (13.3%)	60 (28.6%)	<0.001*
	Type 2	273 (86.7%)	150 (71.4%)	
History of hypertension	Yes	216 (68.6%)	126 (60.0%)	0.044*
	No	99 (31.4%)	84 (40.0%)	
History of dyslipidemia	Yes	239 (75.9%)	135 (64.3%)	0.004*
	No	76 (24.1%)	75 (35.7%)	
History of depression	Yes	123 (39.0%)	57 (27.1%)	0.005*
	No	192 (61.0%)	153 (72.9%)	
History of smoking	Yes	170 (54.0%)	11 (5.2%)	<0.001*
	No	145 (46.0%)	199 (94.8%)	
Physical activity	Yes	141 (44.8%)	129 (61.4%)	<0.001*
	No	174 (55.2%)	88 (38.6%)	

The occurrence of neurological manifestations in both types of diabetes patients revealed that most of the male diabetic patients 227 (72.1%) and female diabetic patients 159 (75.7%) felt tired and weak occasionally, but an insignificant association was observed (p=0.315). Numbness or tingling in the upper and lower extremities was felt by 205 (65.1%) male and 126 (60.0%) female diabetes patients, with an insignificant difference seen among them (p=0.238). Irritability or mood swings were reported by most of the male 216 (68.6%) and 163 (77.6%) female diabetes patients, with a significant relationship noticed (p=0.022). Additionally, there was a significant association observed between both genders in terms of swelling of feet, ankles, wrists, fingers, and eyes (p=0.042), disorientation or trouble focusing (p=0.040), burning sensation in feet or legs (p=0.012), and muscle pain or cramps in legs or feet (p=0.016). Likewise, an insignificant relation was seen between genders with respect to excessively sensitive feet on touch (p=0.471), vision troubles (p=0.217), and cold sweating (p=0.139), as shown in Table 3.

**Table 3:** The distribution of neurological manifestations in type 1 and 2 diabetic patients with respect to gender

Variables		Male	Female	p-value
		n (%)	n (%)	
Feeling tired and weak occasionally	Yes	227 (72.1%)	159 (75.7%)	0.351
	No	88 (27.9%)	51 (24.3%)	
Numbness or tingling in the lower and upper extremities	Yes	205 (65.1%)	126 (60.0%)	0.238
	No	110 (34.9%)	84 (40.0%)	
Irritability or mood swings	Yes	216 (68.6%)	163 (77.6%)	0.022*
	No	99 (31.4%)	47 (22.4%)	
Swelling of feet ankles hands and eyes	Yes	187 (59.4%)	143 (68.1%)	0.042*
	No	128 (40.6%)	67 (31.9%)	
Disorientation or trouble focusing	Yes	109 (34.6%)	55 (26.2%)	0.040*
	No	206 (65.4%)	155 (73.8%)	
Burning pain in legs or feet	Yes	157 (49.8%)	128 (61.0%)	0.012*
	No	158 (50.2%)	82 (39.0%)	
Excessively sensitive feet on touch	Yes	68 (21.6%)	51 (24.3%)	0.471
	No	247 (78.4%)	159 (75.7%)	
Muscle pain or cramps in legs or feet	Yes	266 (84.4%)	192 (91.4%)	0.016*
	No	49 (15.6%)	18 (8.6%)	
Vision troubles	Yes	149 (47.3%)	87 (41.4%)	0.217
	No	166 (52.7%)	123 (58.6%)	
Cold sweating	Yes	173 (54.9%)	129 (61.4%)	0.139
	No	142 (45.1%)	81 (38.6%)	

The association of neurological manifestations in both types of diabetes patients with comorbidities revealed that irritability or mood swings, swelling of feet, ankles, hands, and eyes, disorientation or trouble focusing, excessively sensitive feet to touch, muscle pain or cramps in legs or feet, vision troubles, and cold sweating were significantly influenced by the presence of hypertension (p<0.05). Additionally, dyslipidemia was significantly associated with feeling tired and weak occasionally, disorientation or trouble focusing, burning pain in legs and feet, vision troubles, and cold sweating (p<0.05), as presented in Table 4.

**Table 4:** The association of neurological manifestations in type 1 and 2 diabetes patients with hypertension and dyslipidemia *p-value significant as <0.05

Variables		Hypertension Yes, n(%)	Hypertension No, n(%)	p-value	Dyslipidemia Yes, n(%)	Dyslipidemia No, n(%)	p-value
Feeling tired and weak occasionally	Yes	244(71.3%)	142(77.6%)	0.122	289(77.3%)	97(64.2%)	0.002*
	No	98(28.7%)	41(22.4%)		85(22.7%)	54(35.8%)	
Numbness or tingling in the lower and upper extremities	Yes	206(60.2%)	125(68.3%)	0.068	239(63.9%)	92(60.9%)	0.522
	No	136(39.8%)	58(31.7%)		135(36.1%)	59(39.1%)	
Irritability or mood swings	Yes	222(64.9%)	157(85.8%)	<0.001*	267(71.4%)	112(74.2%)	0.520
	No	120(35.1%)	26(14.2%)		107(28.6%)	39(25.8%)	
Swelling of feet ankles hands and eyes	Yes	235(68.7%)	95(51.9%)	<0.001*	233(62.3%)	97(64.2%)	0.677
	No	107(31.3%)	88(48.1%)		141(37.7%)	54(35.8%)	
disorientation or trouble focusing	Yes	123(36.0%)	41(22.4%)	0.002*	127(34.0%)	37(24.5%)	0.034*
	No	219(64.0%)	142(77.6%)		247(66.0%)	114(75.5%)	
Burning pain in legs or feet	Yes	181(52.9%)	104(56.8%)	0.392	215(57.5%)	70(46.4%)	0.021*
	No	161(47.1%)	79(43.2%)		159(42.5%)	81(53.6%)	
Excessively sensitive feet on touch	Yes	99(28.9%)	20(10.9%)	<0.001*	88(23.5%)	31(20.5%)	0.457
	No	243(71.1%)	163(89.1%)		286(76.5%)	120(79.5%)	
Muscle pain or cramps in legs or feet	Yes	281(82.2%)	177(96.7%)	<0.001*	332(88.8%)	126(83.4%)	0.098
	No	61(17.8%)	6(3.3%)		42(11.2%)	25(16.6%)	
Vision troubles	Yes	175(51.2%)	61(33.3%)	<0.001*	200(53.5%)	36(23.8%)	<0.001*
	No	167(48.8%)	122(66.7%)		174(46.5%)	115(76.2%)	
Cold sweating	Yes	210(61.4%)	92(50.3%)	0.014*	232(61.5%)	72(47.7%)	0.004*
	No	132(38.6%)	91(49.7%)		144(38.5%)	79(52.3%)	

The association of neurological manifestations in diabetes patients with type and duration of diabetes revealed that type 2 diabetes patients had a significantly increased probability of irritability or mood swings, swelling of feet, ankles, hands, and eyes, disorientation or trouble focusing, excessively sensitive feet on touch, muscle pain or cramps in legs or feet, vision troubles, and cold sweating as compared to type 1 diabetes patients (p<0.05). Moreover, there was a significant difference observed in neurological manifestation in diabetes patients with respect to the duration of diabetes (p<0.05), as presented in Table 5.

**Table 5:** The association of neurological manifestations in type 1 and 2 diabetes patients with type and duration of diabetes DM: diabetes mellitus *p-value significant as <0.05

Variables		Type 1 DM, n(%)	Type 2 DM, n(%)	p-value	Duration of Diabetes			p-value
					< 1 year, n(%)	​​​​​​​1-5 Years, n(%)	>5 years, n(%)	
Feeling tired and weak occasionally	Yes	76(74.5%)	310(73.3%)	0.801	113(84.3%)	171(66.8%)	102(75.6%)	0.001*
	No	26(25.5%)	113(26.7%)		21(15.7%)	85(33.2%)	33(24.4%)	
Numbness or tingling in the lower and upper extremities	Yes	64(62.7%)	267(63.1%)	0.944	91(67.9%)	146(57.0%)	94(69.6%)	0.020*
	No	38(37.3%)	156(36.9%)		43(32.1%)	110(43.0%)	41(30.4%)	
Irritability or mood swings	Yes	58(56.9%)	321(75.9%)	<0.001*	102(76.1%)	171(73.3%)	104(77.0%)	0.070
	No	44(43.1%)	102(24.1%)		32(23.9%)	83(73.3%)	31(23.0%)	
Swelling of feet ankles hands and eyes	Yes	54(52.9%)	276(65.2%)	0.021*	86(64.2%)	146(57.0%)	98(72.6%)	0.010*
	No	48(47.1%)	147(34.8%)		48(35.8%)	110(43.0%)	37(27.4%)	
disorientation or trouble focusing	Yes	15(14.7%)	149(35.2%)	<0.001*	10(7.5%)	78(30.5%)	76(56.3%)	<0.001*
	No	87(85.3%)	274(64.8%)		124(92.5%)	178(69.5%)	59(43.7%)	
Burning pain in legs or feet	Yes	60(58.8%)	225(53.2%)	0.305	65(48.5%)	142(55.5%)	78(57.8%)	0.271
	No	42(41.2%)	198(46.8%)		69(51.5%)	114(44.5%)	57(42.2%)	
Excessively sensitive feet on touch	Yes	15(14.7%)	104(24.6%)	0.032*	15(11.2%)	79(30.9%)	25(18.5%)	<0.001*
	No	87(85.3%)	319(75.4%)		119(88.8%)	177(69.1%)	110(81.5%)	
Muscle pain or cramps in legs or feet	Yes	73(71.6%)	385(91.0%)	<0.001*	117(87.3%)	233(91.0%)	108(80.0%)	0.008*
	No	29(28.4%)	38(9.0%)		17(12.7%)	23(9.0%)	27(20.0%)	
Vision troubles	Yes	30(29.4%)	206(48.7%)	<0.001*	25(18.7%)	125(48.8%)	86(63.7%)	<0.001*
	No	72(70.6%)	217(51.3%)		109(81.3%)	131(51.2%)	49(36.3%)	
Cold sweating	Yes	30(29.4%)	272(64.3%)	<0.001*	42(31.3%)	173(66.8%)	87(64.4%)	<0.001*
	No	72(70.6%)	151(35.7%)		92(68.7%)	83(33.2%)	48(35.6%)	

As far as smoking and socioeconomic status are concerned, a significant association was noticed in most of the neurological features in diabetes patients with respect to smoking and socioeconomic status (p<0.05), as presented in Table 6.

**Table 6:** The association of neurological manifestations in type 1 and 2 diabetes patients with smoking and socioeconomic status *p-value significant as <0.05

Variables		Smoking Yes , n(%)	Smoking No, n(%)	p-value	Socio economic status			p-value
					Low, n(%)	Middle, n(%)	High, n(%)	
Feeling tired and weak occasionally	Yes	138(76.2%)	248(72.1%)	0.306	51(59.3%)	272(87.2%)	63(49.6%)	<0.001*
	No	43(23.8%)	96(27.9%)		35(40.7%)	40(12.8%)	64(50.4%)	
Numbness or tingling in the lower and upper extremities	Yes	129(71.3%)	202(58.7%)	0.005*	28(32.6%)	212(67.9%)	91(71.7%)	<0.001*
	No	52(28.7%)	142(41.3%)		58(67.4%)	100(32.1%)	36(28.3%)	
Irritability or mood swings	Yes	113(62.4%)	266(77.3%)	<0.001*	81(94.2%)	224(71.8%)	74(58.3%)	<0.001*
	No	68(37.6%)	78(22.7%)		5(5.8%)	88(28.2%)	53(41.7%)	
Swelling of feet ankles hands and eyes	Yes	115(63.5%)	125(62.5%)	0.815	58(67.4%)	193(61.9%)	79(62.2%)	0.628
	No	66(36.5%)	129(37.5%)		28(32.6%)	119(38.1%)	48(37.8%)	
disorientation or trouble focusing	Yes	79(43.6%)	85(24.7%)	<0.001*	12(14.0%)	116(37.2%)	36(28.3%)	<0.001*
	No	102(56.4%)	259(75.3%)		74(86.0%)	196(62.8%)	91(71.7%)	
Burning pain in legs or feet	Yes	89(49.2%)	196(57.0%)	0.088	71(82.6%)	145(46.5%)	69(54.3%)	<0.001*
	No	92(50.8%)	148(43.0%)		15(17.4%)	167(53.5%)	58(45.7%)	
Excessively sensitive feet on touch	Yes	36(19.9%)	83(24.1%)	0.270	16(18.6%)	83(26.6%)	20(15.7%)	0.030*
	No	145(81.1%)	261(75.9%)		70(81.4%)	229(73.4%)	107(84.3%)	
Muscle pain or cramps in legs or feet	Yes	136(75.1%)	322(93.6%)	<0.001*	75(87.2%)	275(88.1%)	108(85.0%)	0.677
	No	45(24.9%)	22(6.4%)		11(12.8%)	37(11.9%)	19(15.0%)	
Vision troubles	Yes	76(42.0%)	160(46.5%)	0.322	47(54.7%)	143(45.8%)	46(36.2%)	0.026*
	No	105(58.0%)	184(53.5%)		39(45.3%)	169(54.2%)	81(63.8%)	
Cold sweating	Yes	84(46.4%)	218(63.4%)	<0.001*	54(62.8%)	196(62.8%)	52(40.9%)	<0.001*
	No	97(53.6%)	126(36.6%)		32(37.2%)	116(37.2%)	75(59.1%)	

## Discussion

DM is an endocrine disorder that is caused by insufficient production of insulin or by improperly functioning insulin signaling that affects glucose tolerance as well as the metabolism of carbohydrates, proteins, and fats [19]. Although cranial nerves may be implicated, diabetic neuropathy primarily affects the upper and lower extremities in a symmetrical and bilateral distribution. Peripheral symmetrical polyneuropathy is hence the most typical manifestation. DPN is more common in type 2 diabetes patients and often develops after the age of 50 [20]. Therefore, this study demonstrated neurological features in patients with type 1 and 2 DM.

According to research by Ziegler et al., type 2 diabetes women are more likely than men to experience painful DPN [21]. Additionally, it has been demonstrated in another study that patients with painful DPN are more likely to involve people with poor education, those with longstanding uncontrolled DM, and those who are 50 years of age or older [14]. Algeffari et al. stated that 35% of diabetic patients had painful DPN in their study [15]. The present study was in agreement with the above-reported studies and indicated that most of the affected patients had type 2 diabetes, with a male predominance of 273 (86.7%) with a significant difference between both genders (p<0.001).

Similarly, a study conducted in Saudi Arabia investigated 285 participants. There were 58.9% type 2 diabetes, 41.1% type 1 diabetes, and 71.9% who received therapy with insulin injections. Of the participants, 129 (45.3%) were men and about 156 (54.7%) were women. Most of the patients (51.1%) were between the ages of 45 and 64, 77.9% did not smoke, and 79.3% had a low education level. Around 33.5% of the people who experienced neuropathic pain had type 1 diabetes. Out of the total patients with diabetes mellitus, 66.5% had type 2 diabetes (P <0.001). Female patients were more likely to have this persistent uncomfortable sensation or tenderness (40.8%) when they touched their feet or engaged in walking-related activities, whereas male patients did not experience these symptoms. Approximately 2.5% of men and 26.2% of women have persistent burning discomfort in their legs or feet. The prevalence of persistent numbness and decreased sensation was low in men (1.3%) and high in women (19.4%). Additionally, compared with 30.4% of females, 8.9% of males reported persistent prickling or burning sensations. Females were more susceptible to developing neuropathic symptoms than males, with a statistically significant difference noticed between both genders (p<0.001) [22]. The present study was inconsistent with the above study and reported that in most of the patients with neuropathic pain, 273 (86.7%) males and 150 (71.4%) females had type 2 diabetes with a significant difference seen between both genders (p<0.001). Concerning neurological symptoms, most of the symptoms were significantly higher in female subjects than in males, such as irritability, mood swings, swelling, muscular pain, burning pain in legs and feet, and confusion (p<0.05). 

According to prior epidemiological research, the significant risk factors for DPN are the duration of diabetes and increased blood glucose level [23-25]. It was reported by studies that conventional cardiovascular causative factors, such as hypertension, smoking, dyslipidemia, and a greater BMI, have also been linked to problems related to diabetes, besides neuropathy [25,26]. These findings were not corroborated with those of another study that identified depressive symptoms as a newly developed causative factor for DPN. According to their findings, Type 2 diabetes patients with depression increased their chance of developing DPN, regardless of sociodemographic characteristics, including the period of diabetes, HbA1c level, blood pressure, BMI, and the possibility of heart disease [27]. These findings were in accordance with the present study and indicated that the existence of comorbidities such as hypertension, depression, dyslipidemia, inadequate physical activity, duration along with the type of diabetes, socioeconomic status, and smoking influenced most of the neurological manifestations in diabetic patients.

The clinical burden of diabetes and its consequences are always rising, and risk factors like obesity are on the rise everywhere. However, the development of various innovative medications to treat the disease has resulted in therapeutic advancement. Recombinant insulin, innovative medications, and improved treatment regimens all aid in establishing better glycemic control, which stops or delays the advancement of neuronal alterations in DM. However, there is ample opportunity for further research in this domain, particularly in relation to medications that prevent and treat the neurological side effects of DM.

Our study has some limitations, including limited sample size and a lack of long-term follow-up. Moreover, we did not evaluate the risk factors for diabetic neuropathy because this was a cross-sectional study. Although it was a multicenter study, the sample represented a wider area of the population, but the study duration was only eight months, which limited the overall sample size. Similarly, although we sought the association of some associated factors with neurological manifestations in DM, proper evaluation of risk factors requires a different study design (prospective cohort study) with a long follow-up.

## Conclusions

This study concluded that the prevalence of neurological manifestations was high among diabetic patients. Most of the neurological symptoms were significantly more pronounced in female diabetic patients. Moreover, the duration and type of DM (type 2) were significantly associated with most neurological manifestations. Similarly, hypertension, dyslipidemia, and smoking also influenced some neurological manifestations in DM. Neurological symptoms that are painful have a detrimental effect on patients' living standards and may place a significant financial strain on the healthcare system. Proper glycemic management and lifestyle changes are important to prevent the condition from worsening.
